# The Influence of Depression on Cognitive Control: Disambiguating Approach and Avoidance Tendencies

**DOI:** 10.1371/journal.pone.0143714

**Published:** 2015-11-25

**Authors:** He Huang, Javier Movellan, Martin P. Paulus, Katia M. Harlé

**Affiliations:** 1 Department of Cognitive Science, University of California San Diego, La Jolla, California, United States of America; 2 Machine Perception Lab, University of California San Diego, La Jolla, California, United States of America; 3 Department of Psychiatry, University of California San Diego, La Jolla, California, United States of America; 4 Laureate Institute for Brain Research, Tulsa, Oklahoma, United States of America; Radboud University Nijmegen, NETHERLANDS

## Abstract

Dysfunctions of approach and avoidance motivation play an important role in depression, which in turn may affect cognitive control, i.e., the ability to regulate thoughts and action to achieve internal goals. We use a novel experimental paradigm, i.e. a computer simulated driving-task, to study the impact of depression on cognitive control by measuring approach and avoidance actions in continuous time. In this task, 39 subjects with minimal to severe depression symptoms were instructed to use a joystick to move a virtual car as quickly as possible to a target point without crossing a stop-sign or crashing into a wall. We recorded their continuous actions on a joystick and found that depression 1) leads to further stopping distance to task target; and 2) increases the magnitude of late deceleration (avoidance) but not early acceleration (approach), which was only observed in the stop-sign condition. Taken together, these results are consistent with the hypothesis that depressed individuals have greater avoidance motivation near stopping target, but are minimally affected by approach motivation.

## Introduction

Depressive pathology has been linked to executive deficits, including impaired cognitive control [1], which can be defined as the ability to regulate thoughts and action to achieve internal goals [2]. Such impairments are evidenced in a variety of complex goal-directed tasks. For instance, depression seems to affect how individuals process sensory information (e.g., sensitivity to mood-congruent/negatively valenced information) in cognitive control tasks, as well as how they dynamically plan and implement actions in various goal-directed tasks. For example, clinically depressed subjects show motor slowing and deficits in the programming of movement velocity [3]. However, the effect of depression on the implementation of goal-directed actions remains poorly understood, despite important implications for everyday functioning. Mood-driven cognitive control deficits are indeed likely to have deleterious consequences on physical and mental health as they may hinder the degree to which complex decision-making tasks can be accurately and quickly executed on a daily basis, e.g., during driving, shopping, social interactions, etc. Moreover, due to the complex nature of depressive symptoms and related neural systems [4–5], evidence of cognitive control deficits in depression based on standard cued response tasks is at best mixed [6], suggesting a need for more sensitive behavioral paradigms to assess cognitive control in depression.

Motivation plays an important role in cognitive control [7]. Motivation deficits in depressed individuals may lead to impaired performance in cognitive tasks. To understand the deficits of cognitive control in depressed individuals, it is important to examine the motivational processes that orienting the control process. Affective states not only affect cognitive but also motivational processes. For example, sad affect in depressed individuals may enhance mood-congruent information processing [8–9], which may result in reduced motivation to pursue rewards. Moreover, relative to healthy controls, depressed individuals pay less attention to positive stimuli [8] and report lower positive affect in response to positive stimuli [10]. Finally, students with a range of self-reported depressed mood were slower to approach positive social cues [11]. Anhedonia (the decreased capability to experience pleasure and seek reward) is another essential symptom of Major Depressive Disorder (MDD; DSM-V; American Psychiatric Association, 2013) and is a core feature of reward-processing deficits in depression. It has been suggested that anhedonia might reduce the motivation to pursue future rewards and engage in pleasurable activities [12]. That is, a decreased ability to experience pleasure may reduce the incentive to act to seek pleasure or reward, leading to further decrease in positive affect and thus perpetuating reward-based motivational deficits in depression. Treadway & Zald 2011 [13] argued that depression may not only be affected by ‘consummatory anhedonia’, i.e. the deficits in the hedonic response to rewards, but also by ‘motivational anhedonia’, i.e. diminished motivation to pursue rewards. For instance, in a signal detection task under different monetary payoff conditions, clinically depressed individuals were shown to be less discriminative of and also less sensitive to monetary rewards, which lead to lower performance accuracy [14]. These reward-seeking deficits appear in the context of decreased recruitment of the nucleus accumbens and prefrontal cortex (PFC) upon exposure to rewards, which is believed to reflect impairment in attentional switching, regulatory processes [15–16] and in flexibility to alter reward-seeking behavior [17].

While motivational research often focuses on approach deficits in depression, a disturbance in avoidance processes may be equally important and relevant to cognitive control performance among depressed individuals [18]. For example, depressed patients learn faster to avoid risky gambles [19], and demonstrate faster motor response to withdraw from negative stimuli such as negative faces [20]. It has also been suggested that one of the characteristics of depression is the increased difficulty to disengage from negative material [21–22]. Such a negative attention bias may lead to deploying greater cognitive effort to move away from undesired states (avoidance goals; [23]). This has been reported in a variety of studies, with evidence suggesting that depression is associated with more avoidant schemas and emotions [24]. Overall, these findings suggest that depression may result in an attenuated motivation to approach reward as well as greater sensitivity to and a higher motivation to avoid punishment. However, few studies [25] have attempted to quantify these motivational biases in depressed individuals, particularly in terms of their influence on motor-control in complex goal-directed tasks.

### The Present Study

The research reviewed above, as well as neural studies of frontal asymmetry, have highlighted the mapping of psychological motivational states to basic actions tendencies in healthy and clinical populations, such that higher avoidance and lower approach motivation observed in depression and negative emotionality have been associated with both decreased left-frontal and increased right frontal activity, respectively associated with reduced approaching actions and increased avoidant actions (e.g., joystick pulling/pushing to increase or reduced distance from various stimuli; [19, 26–28]). Based on this research, we hypothesized that such motivational biases in depression and sad affect might influence cognitive control in a paradigm involving goal-directed actions. This hypothesis was based on evidence that emotion, including non-task related affective states, can influence people’s goals and planning, and produce tangible behavioral biases [29–31]. According to the empirically validated affect infusion model (AIM; [30]), emotion, including their underlying motivational core (approach vs. avoidance), conveys information, which may be automatically integrated in a wide range of higher-order executive processes (e.g., cognitive control) through interoception (i.e., by conveying information about ones’ valuation of /disposition towards task-related goals). We would therefore expect that broad motivational tendencies intrinsic to an individual’s psychological state (e.g., depressed mood), such as approach and avoidance tendencies, might similarly carry over into cognitive control by enhancing motivationally congruent goal-directed tasks, and/or dampening motivationally incongruent actions.

To quantify such potential biases, and to gain a more precise understanding of how depression and its underlying motivational biases may contribute to cognitive control dysfunction in real life settings, we designed a computer joystick-operated driving task allowing us to distinctively measure both approach and avoidance tendencies in continuous time. Different from traditional paradigms with explicit affective approach/avoidance motivation, i.e., approach for rewards and avoidance for punishments, here we measure approach-avoidance motivation using continuous actions without explicit feedback (e.g. no points or any measure of performance evaluation). Therefore, the degree of approach or avoidance is defined by the action-related behavioral parameters rather than a response to a reward or punishment and could represent general approach/avoidance tendency dysfunctions in depression. In this task, subjects are instructed to drive a car to a target as quickly as possible, and stop as closely as possible without crossing the target line. The ‘goal’ in the task is to *approach* the instructed location while *avoiding* crossing the stopping line. Higher acceleration indicates a stronger approaching action (i.e. pushing forward the joystick) relative to the initial resting position, while a higher deceleration indicates the avoidant action of slowing down (i.e. pulling back the joystick). According to the AIM, in such simulated driving context, approach and/or avoidance motivational bias may manifest by enhancing motivationally congruent actions through the motor control demands of the task (i.e., approaching a target vs. avoiding to go pass the target line, respectively), which would translate in terms of acceleration/deceleration. Thus higher avoidance motivational tendency in depressed individuals may result in stronger avoidant actions (e.g., braking through joystick pull), whereas their lower approach motivation could impact approach actions (e.g., accelerating though joystick push).

## Materials and Methods

### Participants

Thirty-nine college students (age range: 18–22 years old, 23 females /16 males) participated in this study (approved by the Human Research Protections Program at San Diego State University). They were recruited from San Diego State University through an online system as part of Psychology 101 class during the spring of 2013. They were contacted and scheduled for an experiment session during winter quarter 2013. All participants signed informed consent, and were compensated $25 and 2.5 course credits for completing the study.

Before subjects performed the experimental task, they were administered the Beck Depression Inventory (BDI-II; [32]) to use as a current index of depression severity. Participants’ BDI scores ranged from 0 to 40 (mean = 13.5, std = 10.77; median = 10). For the following analyses, we grouped subjects (Table 1) by the severity of their depression symptoms based on their BDI score (cutoff based on [32]). The final groups included 15 non-depressed subjects with BDI≤8 (Non Dep), 11 mildly depressed subjects with 9 ≤ BDI ≤ 14(Mild Dep), and 13 moderately to severely depressed subjects with BDI≥15 (Mod-Sev Dep). The groups did not differ in age (F (2, 36) = 1.35, *p* = .27), or gender (chi-square = 4.06, *p* = .13) among groups.

**Table 1:** Demographic and BDI comparison across the three depression severity groups.

Characteristics	Non-dep	Mild Dep	Mod-Sev Dep
N	15	11	13
Age mean (std)	19.13 (1.06)	19 (1.18)	20.4 (3.75)
Sex (male%)	60%	36%	23%
BDI mean (std)	4.07 (2.6)	10.7 (1.57)	26.75 (8.50)

### Driving Task

**Fig 1:**
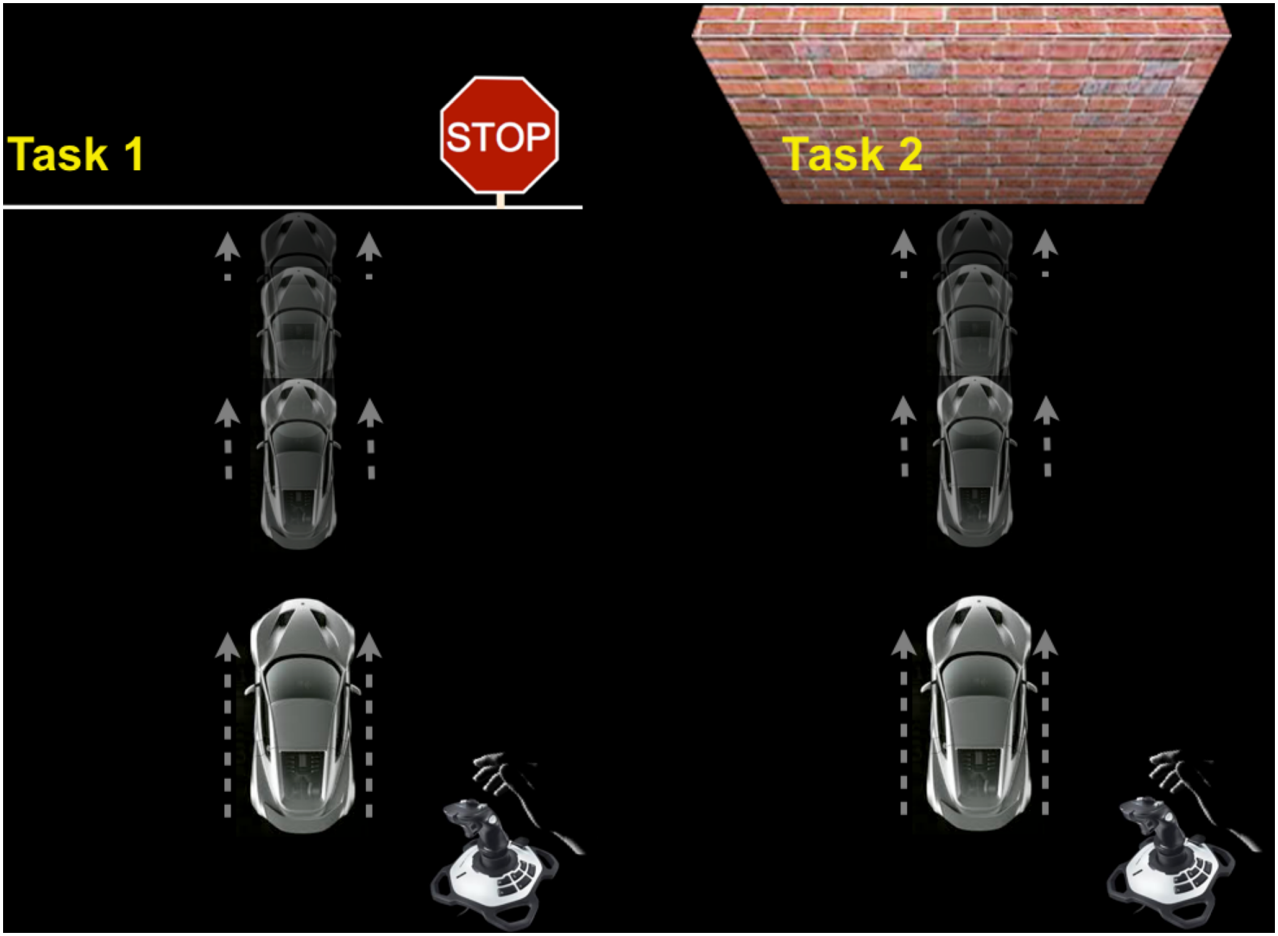
Experiment paradigm. In Task1 (stop-sign), subjects were instructed to drive the car to the stop-sign as quickly as possible and stop as close as possible to the sign without crossing the white line. In Task 2 (wall), subjects received the same instructions as in task 1, with the addition that they could not crash into the wall (instead of not crossing the stop-sign). Both tasks have a fixed time window of 6 seconds.

The experiment was run on a 15.4-inch laptop (resolution: 1440 x 900), with the tasks programed using Psychtoolbox-3 in Matlab R2010a. The experiment was comprised of two conditions (Task 1: stop-sign; Task 2: wall). In each trial, subjects were instructed to drive a virtual car on a computer screen from an initial position to either a stop-sign or a wall (equal distance: 10.62 cm/465 pixels) by pushing or pulling a joystick (Logitech Extreme 3D Pro) to control the acceleration and the deceleration of the car (Fig 1). They were instructed to drive as quickly as possible and stop as close to the stop-sign or the wall as possible without crossing the stop-sign or crashing into the wall, respectively. Each trial had a fixed time-window of 6 seconds. Participants completed two blocks of 15 trials for each condition in the following order: Task1, Task2, Task1, Task 2. Task 1 presented prior to Task 2 is designed to avoid the influence from the impact of the wall condition (in which subjects cannot pass the wall) on the stop-sign condition (in which subjects can pass the stop-sign). Specifically, subjects starting with the wall condition, i.e., in which they are constrained to stop before the wall, may be more likely to benefit from a learned motor trajectory, which would in turn reduce the likelihood to cross the stop line in the stop-sign condition (since stop-sign and wall have the same distance from the starting point). However, subjects starting with the stop-sign condition, in which they are *not* constrained to stop before the stop target line, can still show learning of and adaptation to a new, more constrained, motor trajectory in the wall condition.

The car dynamics adhered to the standard second order Newtonian differential equations of motions with viscous friction. The force applied to the car at every point in time was proportional to the deviation of the Joystick from the resting point. Forward joystick action (joystick position > resting point) will lead to the acceleration of the car speed, while backward joystick action (joystick position < resting point) will lead to the deceleration of the car speed. The position of the joystick directly controls the magnitude of the acceleration or the deceleration (See S1 File in Supporting Information for more detail).

### Data Analytic Approach

The two main dependent variables were participants’ stopping distance, i.e. distance from stop- sign /wall in pixels and participants’ continuous acceleration/deceleration action through joystick control (measured as joystick position). The first variable corresponds to car position when T = 6s, i.e., trial time window, and the second one to recorded continuous joystick action for acceleration/deceleration. Based on the observed trajectories, further analyses were performed on individuals’ maximum acceleration during the initial trial phase (i.e., max acceleration during the first 200 pixels) and their individual maximum deceleration in the late trial/close to target phase (i.e., max deceleration during the last 200 pixels).

To analyze these data, a linear mixed model (LMM) was fitted for each main dependent variable in R, with subject modeled as a random effect and depression severity group as well as condition as fixed effects. To ensure participants’ performance was stable and avoid confounding factors such as subjects’ learning curve in the task, only data collected in the second block for each condition were included in the analyses. We first assessed for any group differences in stopping distance. If differences were observed, we followed up by investigating when this difference happened within a trial epoch, and what actions caused this difference. For significance tests of main effects and interactions, we report change in log likelihood ratio (modeled by a chi-square distribution). We also provide the model coefficients of interest with corresponding p values and Cohen’s d or D to indicate the effect size.

## Results

### Depression is associated with more remote stopping position

We first examined the effect of depression group (i.e., Non Dep, Mild Dep and Mod-Sev Dep) and experimental condition (i.e., Stop sign, Wall) on stopping distance. There was a significant group main effect (chi-square = 12.60, df = 2, *p* = .002) and significant interaction between group and condition (chi-square = 12.98, df = 4, *p* = .011) while no main effect of condition was observed (chi-square = 2.86, df = 1, p > .05).

**Fig 2:**
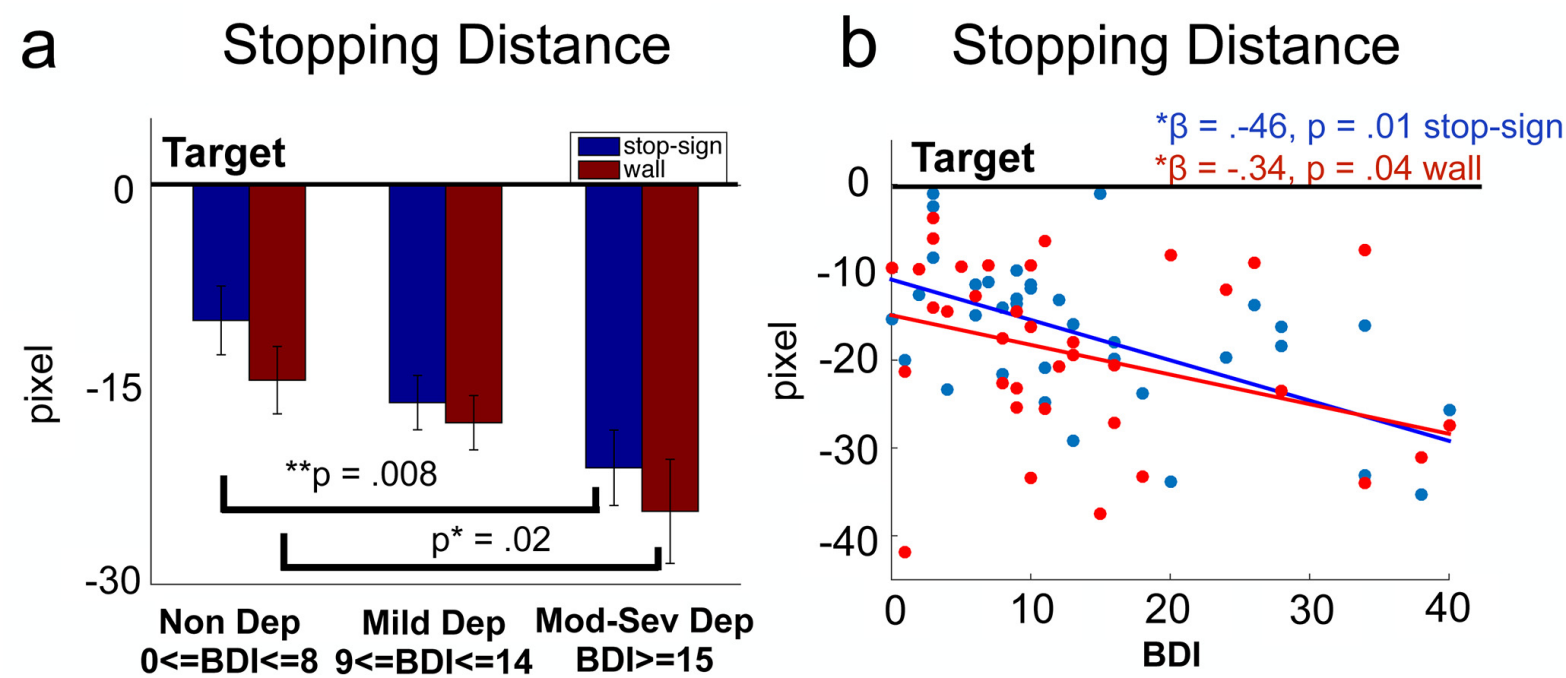
The influence of depressed mood on stopping distance in stop-sign and wall condition. a. Group comparison. P-values are Bonferroni corrected for multiple comparisons. Black line is the target (stop-sign or wall). Blue bars represent the stopping distance of the 3 groups in stop-sign condition and red bars represent that in wall condition, respectively. b. Stopping distance as a function of BDI. Each data point represents each individual’s stopping position relative to the target.

Specifically, in the stop-sign condition, relative to Non Dep individuals, the Mod-Sev Dep group stopped significantly further away from the target (B = -11.08, *p* = .008,D = 1.18, 95% CI [-19.20, -2.96]; Non Dep mean stopping distance = 10.15, Mod-Sev Dep mean stopping distance = 21.23), while the Mild Dep and the Non Dep groups did not differ in stopping distance (*p* >.05, D = .75; see Fig 2a). In the wall condition, relative to Non Dep individuals, the Mod-Sev Dep group also had a significantly further stopping distance (B = -9.86, *p* = .02, D = .89, 95% CI [-17.53, -2.19]; Non Dep mean stopping distance = 14.65, Mod-Sev Dep mean stopping distance = 24.51), while the Mild Dep and the Non Dep groups did not differ in their stopping distance (*p* >.05, D = .39) in the wall condition.

Individual’s average stopping distance was negatively correlated with BDI scores in both stop-sign and wall conditions, indicating that the degree of depression was associated with differences in behavior on this task (Fig 2b). In other words, those individuals with the highest BDI scores stopped furthest away from the target position. This negative correlation was slightly higher in the stop-sign condition (regression coefficient = -.46, *p* = .01,*r* ^2^ = .16) than in the wall condition (regression coefficient = -.34, *p* = .04,*r* ^2^ = .11), but this difference was not statistically significant (chi-square = 3.62, df = 2, *p* = .16). A within-subjects t-test of the stopping distance across conditions did not reveal a significant effect (*p* = .14, D = .52), i.e. subjects did not stop systematically differently during the wall or the stop sign condition.

### Depression affects the magnitude of deceleration (avoidance), but not acceleration (approach)

**Fig 3:**
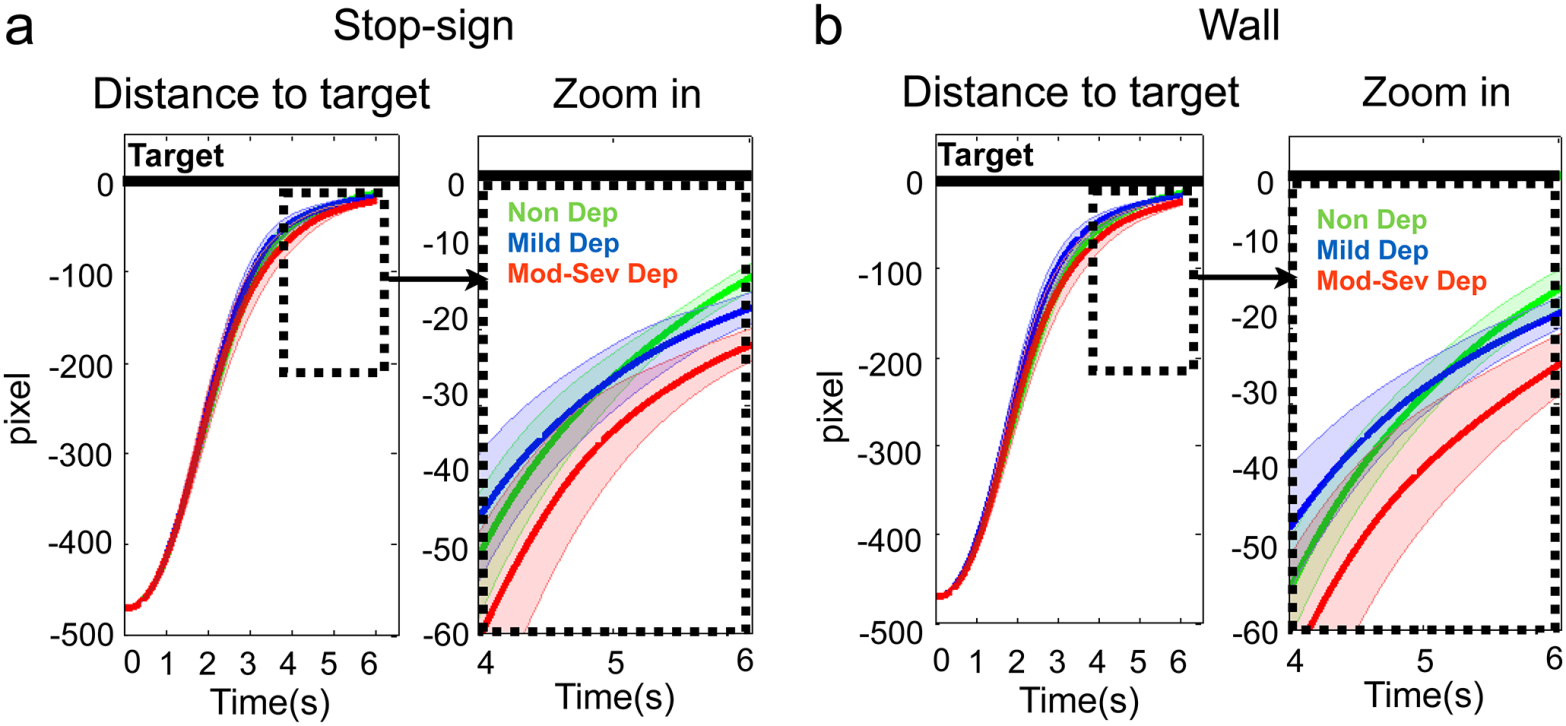
Car distance as a function of time within a trial (6-seconds): a (stop-sign), b (wall). Black solid line: the position of the target (stop-sign or wall). Green solid line: Non Dep. Blue solid line: Mild Dep. Red solid line: Mod-Sev Dep. For each condition, car distance to task target as a function of time is plotted on the left, and zoom-in in the last 2 seconds is plotted on the right.

**Fig 4:**
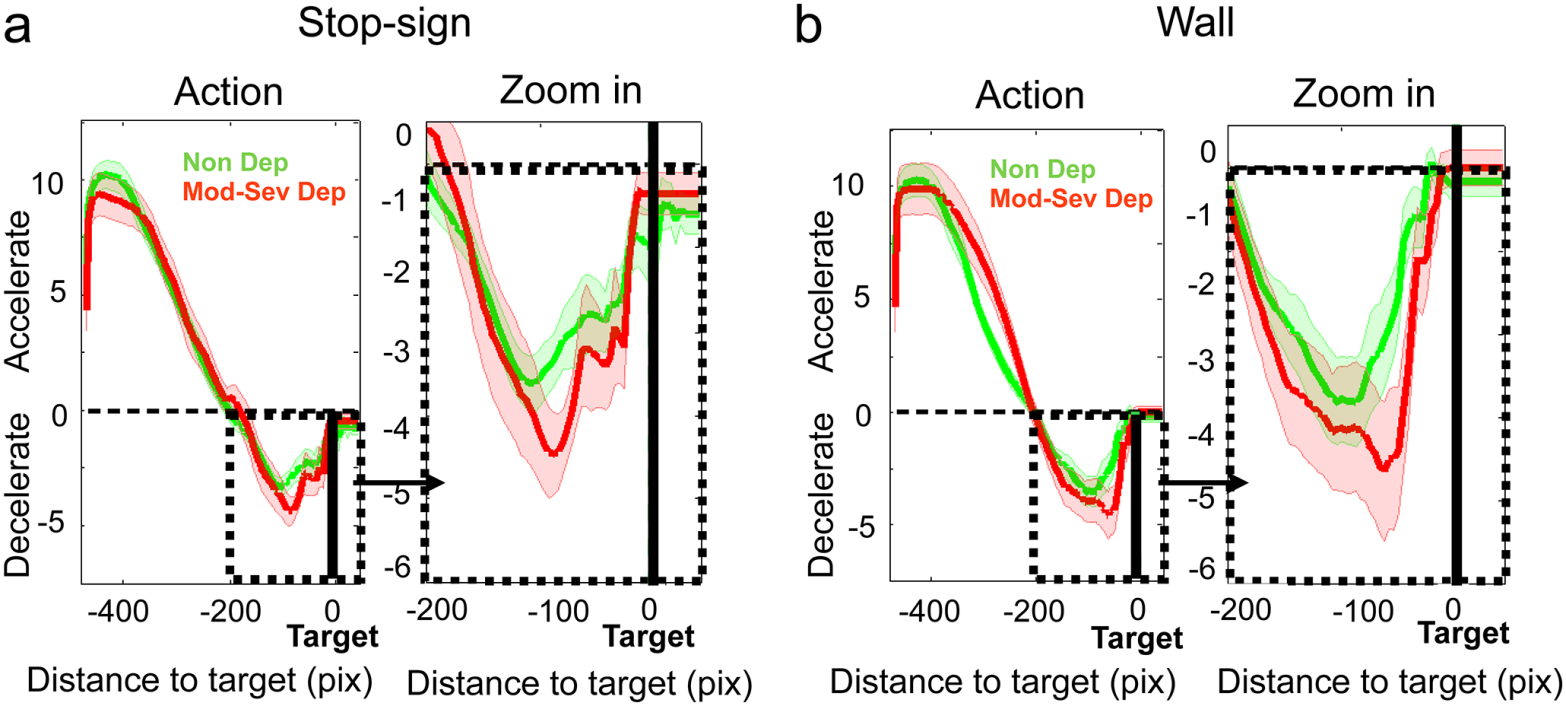
Joystick action as a function of distance to target: a (stop-sign), b (wall). Black solid line: the position of the target (stop-sign or the wall). Green solid line: Non Dep. Red solid line: Mod-Sev Dep. For each condition, joystick action as a function of car distance to target is plotted on the left, and zoom-in in the last 2 seconds is plotted on the right. Positive action (>0) leads to acceleration of the car’s speed, while negative action (<0) leads to deceleration of the car’s speed.

Next, we examined continuous car position between three groups within the 6 seconds trial time window for the stop-sign and wall conditions separately. Fig 3 indicates that car position between Non Dep and Mod-Sev Dep groups started to differ when it was close to the target (3a. stop-sign and 3b.wall), in particular during the last 2 seconds of a trial (Fig 3). It also shows there were no significant position differences between Non Dep and Mild Dep groups throughout the 6-sec trial window, which is consistent with the stopping position results above. To further investigate the cause of this position difference between the Non Dep and Mod-Sev Dep groups, we looked at joystick actions between those the two groups. Action (accelerating if > 0, and decelerating if < 0) is plotted as a function of distance to target in Fig 4.

Maximum Acceleration: there was no significant group main effect (chi-square = 0.10, df = 1, *p* = .75), condition main effect (chi-square = .58, df = 1, *p* = .45), or interaction effect (chi-square = .89, df = 2, *p* = .64) for maximum acceleration (i.e., individuals’ peak acceleration within first 3-seconds trial window) between Non Dep and Mod-Sev Dep groups in the stop-sign and the wall condition.

Maximum Deceleration: We found a significant group effect (chi-square = 17.75, df = 1, *p* < .001) and group by condition interaction (chi-square = 22.58, df = 2, *p* < .001) for maximum deceleration (i.e., individuals’ peak deceleration within last 2-sec trial window). No significant condition main effect was observed (chi-square = .001, df = 1, *p* = .97). More specifically, the Mod-Sev Dep group had overall greater maximum deceleration (i.e., larger dip) relative to the Non Dep group (B = -.97, *p* < .001, 95% CI [-1.41–0.53]). In addition, this group difference was statistically significant in the stop-sign condition (B = -1.47, *p* < .001, 95% CI [-2.06, -0.87]) but not in the wall condition (*p* >.05).

## Discussion

This study aimed to disambiguate the potential impact of approach from avoidance motivational dysfunctions on cognitive control among individuals with depression. Different from traditional approaches using explicit reward/punishment feedback to assess approach/avoidance motivations through discrete actions, we designed a computer-simulated driving-task to examine how the severity of depression (measured with BDI scores) would impact the motivational underpinnings of individuals’ driving behavior through their continuous accelerating and decelerating control without explicit feedback in the experiment. As we outline below, our results are consistent with the hypothesis that individuals with depression engage in stronger avoidance actions while minimally impacting approach-based actions when getting closer to a target. As a consequence, it may negatively impact deceleration and stopping distance. We summarize our results and potential implications below.

Relative to healthy controls, individuals with higher BDI stopped further away from the stop-sign or wall, suggesting they may have a different ‘target’ stopping position. In addition, more severely depressed individuals demonstrated a significantly stronger deceleration when approaching target (i.e. greater avoidant action). These results suggest that higher depression severity may promote stronger proactive avoidance of the target (pulling away) in terms of planning stopping position and adjusting movement closer to end goal. This is consistent with empirical evidence of both heightened sensitivity to and stronger avoidance of negative stimuli in depressed individuals. For instance, depression is associated with increased attention to negative cues in the environment, which has been observed in this population for various cognitive tasks [33], including punishing events such as errors. The latter is demonstrated by stronger amplitudes of the error related negativity (ERN; [34–35]) and increased post-error slowing [36]. Depression has been also associated with faster withdrawing responses from negative stimuli such as negative faces using computer joysticks ([19, 11]).

Alternatively, given evidence of decreased reward-seeking and approach motivation in depression [37], it could be argued that more severely depressed individuals have less motivation to perform the task due to anhedonia [38]. That is, they may be less motivated to be precise about the stopping position, i.e. reduced effort to achieve high accuracy, and as a result stop further away. In addition, their increased stopping distance might be the result of attenuated motor control due to fatigue while still avoiding errors. However, our results show that the high depression group did not differ from healthy controls in terms of their positive control action values (first part of trial epoch), but rather applied higher deceleration when the car was getting closer to the stop sign. Thus, depressed individuals may be less efficient in incorporating environmental cues that indicate increased need for cognitive control (e.g., seeing a stop sign in the distance), which provides evidence for their abnormal pro-active and reactive control processes [1, 39], as found in the classic interference paradigms such as Go/NoGo and Stop-signal tasks.

In non-emotional Go/NoGo tasks, depressed individuals did not differ from healthy controls on Go trials [40], but exhibited an inhibitory control deficit on NoGo trials with increased rates of commission errors [41]. Similarly, without emotional cue or any performance feedback in our task, we showed that there was no significant difference in approaching (which resembles a ‘Go’ signal) between depressed and healthy individuals, whereas the difference appears in the avoidance of a stop- sign. It is important to point out that the stop-sign in our task is not the same as a ‘NoGo’ stimulus. Instead, it acts as a signal indicating the potential for performance errors. The increased stopping distance we observed in depressed individuals may be due to an increased aversive salience of this stimulus. While previous Go/NoGo paradigm focused primarily on discrete decisions with short reaction time frames, our approach provides rich data to examine how action is determined from the state of the environment (e.g. car distance to stop-sign) at each time point. That is, examining the full motor trajectory from continuously action monitoring enables closer inspection of the interaction between Go-activation and NoGo-inhibition in continuous time. The recording of a continuous motor trajectory in effortful cognitive tasks has several advantages over traditional button press responses. First, the trajectory provides a rich behavioral response repertoire, which can be analyzed using sophisticated inverse optimization approaches. Second, approach and avoidance tendencies can be extracted across different time epochs relative to the target. Third, the distance to target provides an objective measure of performance, which can be related to or differentiated from the mood-driven subjective biases. Fourth, modification of the current paradigm can be used to modulate the level of cognitive control necessary for optimal performance. For example, additional stop-signal cues can be provided, which can be used to investigate how stimulus-response (S-R) contingency variations influences motor inhibition [42], and how stop-signal probability affect both proactive and reactive inhibition in a sop-signal anticipation task [43].

Recent neuroimaging studies support the notion that cognitive inefficiency for environmental cues during cognitive control conditions may be due to increased emotional response to these cues, which may result in poorer initial action planning and delayed breaking action when closer to target. For instance, recent studies point to an association between depression severity and ACC activation to interference in standard Stroop paradigms ([44–46]). Such ACC hyperactivity may indicate greater conflict processing and thus noisier, less efficient motor control associated with greater action cost in the close to target range (i.e., delayed implementation of avoidant actions requiring greater late deceleration effort). Moreover, higher sensitivity to punishment and avoidant tendencies in depression has been linked to increased limbic reactivity (particularly the thalamus, amygdala and rostral ACC), thought to reflect stronger bottom-up processing of negative cues [47–49], and to decreased activity in the dorsolateral prefrontal cortex (DLPFC), which is likely to be reflect a reduced ability to disengage from negative stimuli and related avoidant goals [17]. Thus, based on this literature, the present results may point to a stronger cognitive processing of and interference from visual cues that predict the need to implement cognitive control.

Another plausible explanation is that more depressed individuals are lacking fine and/or more precise motor control caused by impaired sensorimotor skills, which would lead to more inaccuracy and variability in stopping positions. That is, while there is somewhat mixed evidence of psychomotor retardation in depression [50–51], higher depression severity could result in slower sensory processing and slower motor initiation. We note, however, that we did not observe slowness in accelerating or decelerating actions in the depressed groups at any points during the trial epoch. It is possible that our findings may only generalize to the population studied here, i.e. college-aged students with a range of self-reported depression symptoms. However, this does not rule out that sensorimotor factors may play a role in more severely, clinically depressed individuals. Therefore, we plan to test the current paradigm in a clinical population with a larger sample size, and also incorporate individual differences in sensorimotor skills in the modeling of how goal setting and reward-processing influence motor control.

Interestingly, while a similar pattern of results was observed in the wall condition (i.e., stronger avoidance effects in more depressed individuals), the group difference was only observed for stopping distance and did not reach statistical significance for late deceleration. This was unexpected given that the wall condition may be perceived as involving a stronger punishment potential (from a sensory standpoint) if the target is missed, which should amplify rather than minimize avoidance. However, this finding needs to be interpreted with caution. First, on closer examination, this lack of significant group difference in the wall condition appears to stem from greater variability of maximum deceleration in the high depression group, who still had greater average peak deceleration than non-depressed subjects. Moreover the average peak deceleration in the high depression group appeared slightly shifted in time to even closer to target, consistent with less efficient motor control implementation, i.e., greater delay of avoidance actions (see Fig 4b). Second, the fixed task order (Stop-sign, Wall, Stop-sign, Wall) used here may have imparted a learning effect and reduced our ability to detect a group difference. Using a novel experimental paradigm to study the condition effect (Stop-sign vs. Wall), we initially designed the task such that the stop-sign and the wall have equal distance from the starting point. It was interesting to find out that the effect of the wall condition is not significant in the current task order. However, it is not that surprising, as subjects may have been over-trained in the stop-sign condition and thus fine-tuned their motor trajectory in the wall condition. It is important for future experiments to keep in mind such motor learning over time. In future studies it may be useful to vary the distances of the stop sign and the wall, and also counter-balance the task orders to eliminate the possible effect of adapted motor trajectory from prior experience. Finally, while our simulated driving task is a new paradigm and we could not directly estimate the effect sizes to be expected, a review of the literature with some form of goal-directed motor dependent measures indicated that likely effect sizes for depressed vs. control group differences ranged from medium to large [50, 52–54]. This suggests that, based on our group sample sizes we were likely underpowered for detecting small and medium effect sizes, which is a limitation of this study.

In conclusion, these results provide empirical evidence of altered motivational influences in motor control performance among individuals with depression. We found that those individuals with the most severe level of depression stopped furthest away from the target and showed the greatest deceleration when approaching the instructed stopping target. Together, these results show that the influence of depression in modulating approach and avoidance motivations and cognitive control may be more fluid and dynamic than expected, with the potential to influence different stages (action planning and execution) of a motor-control task. They further highlight the usefulness of a continuous time analysis as done in the present study. For example, this task can be extended to study more complex ‘driving’ behavior, as recent studies showed that depression may increase the odds of a car accident [55], and reduce driving performance in a driving simulator [53–54]. Finally, our task provides a useful platform to study the precise cognitive processes underlying emotion/cognitive control interactions in depression. Specifically, we plan to use computational models (e.g. control theory) to examine if/how the severity of sensorimotor impairment interacts with motivational deficits in more severely depressed individuals, in particular, to distinguish the effects of sensorimotor speed, goal state (target stopping distance), and motivation underlying a depressive state in goal-directed motor tasks. This research will in turn help to more precisely quantify cognitive control deficits in depression and their associated neural circuitry.

## Supporting Information

S1 Data Data access. (DOCX)

S1 File Car dynamics in the experiment. (DOCX)
