# Supplementary material for: The Influence of Depression on Cognitive Control: Disambiguating Approach and Avoidance Tendencies
Source: PLoS One. 2015 Nov 25;10(11):e0143714. doi: 10.1371/journal.pone.0143714 (PMC4659610; doi:10.1371/journal.pone.0143714)
Supplement: S1 Data — (DOCX) [file pone.0143714.s001.docx]

**S1 Data.**

Data used in the analysis of this paper: http://dx.doi.org/10.5061/dryad.78dg8

(Journal editors and reviewers can find it here: http://datadryad.org/review?doi=doi:10.5061/dryad.78dg8)

For questions regarding this dataset, please contact He Huang (heh001@ucsd.edu).
