# Supplementary material for: The Influence of Depression on Cognitive Control: Disambiguating Approach and Avoidance Tendencies
Source: PLoS One. 2015 Nov 25;10(11):e0143714. doi: 10.1371/journal.pone.0143714 (PMC4659610; doi:10.1371/journal.pone.0143714)
Supplement: S1 File — (DOCX) [file pone.0143714.s002.docx]

**S1 File. Car dynamics in the experiment:**

In our experiment (both stop-sign and wall conditions), the car has a linear dynamic system, which is modeled by the following equation:

d*Xt* = *AXtdt* + *BUtdt,*

in which *Xt* = state [car position, car velocity], *Ut* = control action (acceleration or deceleration, based on joystick position), A = [0 1; 0 -.35] is the dynamic matrix, and B = [0; 0.5] the input matrix. Those valued are chosen such that the car’s velocity is the rate of change of the position:

d*Xt* = *Vtdt* ,

and velocity is controlled by joystick action with the influence of viscosity d*Vt* = -.35*Vtdt*+.5*Utdt*, in which *Ut* is measured through joystick position (ranges from -10 to 10, with 0 being the resting position/no action, positive number indicating accelerating action and negative number indicating decelerating action).

In the reported analysis, we used *Xt* at t = T (T = 6s, trial time window) as the stopping position, maximum *Ut* during the first 200 pixels as participant’s max accelerating action in the during the initial trial phase, and minimal *Ut* during the last 200 pixels as participant’s max decelerating action in the later trial phase.
